# Macrophage Phenotype and Fibrosis in Diabetic Nephropathy

**DOI:** 10.3390/ijms21082806

**Published:** 2020-04-17

**Authors:** Priscila Calle, Georgina Hotter

**Affiliations:** 1 Department of Experimental Pathology, Institut d’Investigacions Biomèdiques de Barcelona-Consejo Superior de Investigaciones Científicas-Institut d’Investigacions Biomèdiques August Pi i Sunyer (IIBB-CSIC-IDIBAPS), Rosselló 161, 7th floor, 08036 Barcelona, Spain; priscila.calle@iibb.csic.es; 2 M2rlab-XCELL, c/ Juan Bravo 10, Bajo, Puerta 2, 28006 Madrid, Spain

**Keywords:** macrophages, diabetic nephropathy, fibrosis

## Abstract

Diabetic nephropathy (DN) is the leading cause of end-stage renal disease globally. The primary initiating mechanism in DN is hyperglycemia-induced vascular dysfunction, but its progression is due to different pathological mechanisms, including oxidative stress, inflammatory cells infiltration, inflammation and fibrosis. Macrophages (Mφ) accumulation in kidneys correlates strongly with serum creatinine, interstitial myofibroblast accumulation and interstitial fibrosis scores. However, whether or not Mφ polarization is involved in the progression of DN has not been adequately defined. The prevalence of the different phenotypes during the course of DN, the existence of hybrid phenotypes and the plasticity of these cells depending of the environment have led to inconclusive results. In the same sense the role of the different macrophage phenotype in fibrosis associated or not to DN warrants additional investigation into Mφ polarization and its role in fibrosis. Due to the association between fibrosis and the progressive decline of renal function in DN, and the role of the different phenotypes of Mφ in fibrosis, in this review we examine the role of macrophage phenotype control in DN and highlight the potential factors contributing to phenotype change and injury or repair in DN.

## 1. Introduction

Diabetic nephropathy (DN) is currently the most prevalent chronic kidney disease and the leading cause of end-stage renal disease in adults [1]. A role for macrophages (Mφ) in both the development and progression of this disease is recognized.

Mφ and renal inflammation are associated with DN as an analysis of renal biopsies in diabetic patients has confirmed the presence of Mφ in both glomeruli and the interstitium at all stages of DN [2,3]. Mφ accumulate in the diabetic kidneys [4,5,6] has been related to renal disease progression [2]. This accumulation correlates strongly with serum creatinine, interstitial myofibroblast accumulation and interstitial fibrosis scores [7,8,9,10,11]. Additionally, in diabetic *db/db* mice, it has been shown that macrophage accumulation and activation provokes glomerular and tubular damage, albuminuria, elevated plasma creatinine, renal fibrosis and kidney expression of Mφ chemokines [12].

Mφ are considered an important source of tumor necrosis factor-alpha (TNF-α) and it is known that this cytokine does play a pivotal role in the development of DN. In this sense, TNF-α levels in kidneys are increased in experimental animal models of DN [13,14] and conditional knockout of TNF-α in Mφ revealed a complete block of TNF-α expression in diabetes-induced models. In addition, deletion of macrophage TNF-α provoked a reduction in hypertrophy, albuminuria and glomerular pathology [15]. Pharmacological inhibition of TNF synthesis reduced the loss of glomerular filtration rate in patients with DN [16] and high TNF receptors are indicative of disease progression in humans with DN [17,18].

The role of fibrosis in the progression of DN has also been recognized appearing to be critical for final progression of DN to kidney failure in diabetic Type 1 and 2 [19]. There is a positive correlation between the grade of fibrosis of the renal cortical interstitium and the serum creatinine concentration at the time of biopsy in patients with DN. This fibrosis appears to be largely due to increase cellular components and Mφ presence, which is followed by an increase in interstitial fibrillary collagen.

Mφ recruitment generates inflammatory cytokines that may stimulate cells to enhance its production or reduce the degradation of matrix proteins [20]. Targeted deletion of the macrophage scavenger receptor-A ameliorated many of the glomerular changes of experimental DN in mice. In these experimental conditions, Mφ infiltration was decreased, proinflammatory genes were suppressed and attachment of monocytes to type IV collagen was reduced [21]. In addition, glomerular and tubulointerstitial cells produce a multitude of inflammatory mediators in the diabetic milieu, especially as injury proceeds, which can augment inflammatory damage and modify Mφ behavior in fibrosis.

Given the strong associations between fibrosis and the progressive decline of renal function in DN, and the recognized role of Mφ as inductors of fibrosis, in this review, we discuss the role of Mφ in both the development and progression of fibrosis in DN. We examine the role of Mφ phenotype in fibrosis development and highlight its implications for new therapeutic strategies. 

## 2. Macrophage Phenotype and Fibrosis

Fibrosis is a process characterized by excessive deposits of extracellular matrix that leads to the replacement of functional parenchyma by fibrotic tissue [22]. Renal fibrosis is the common pathological process in chronic kidney disease, despite the underlying cause, in which kidney gradually lost its ability to repair as a result of ongoing tissue injury and inflammation [23]. However, renal fibrosis is a multifactorial and dynamic process that carries many cellular events in response to the injurious stimuli. Within the several cells types that are implicated in the pathogenesis of renal fibrosis, Mφ gains attention due to the potential therapeutic approaches mediated by cell therapy transfer. These highly heterogeneous cells belong to the mononuclear phagocyte system and are virtually present in all tissues as monocyte-derived Mφ from bone marrow and/or as tissue-resident Mφ that arise from embryonic precursors; the latter self-renew in situ independent of circulating monocytes [24,25]. Mφ has the ability to eliminate pathogens, apoptotic cells or any other foreign body through phagocytosis or T cells activation, which can either contribute to tissue repair or promote further damage. These contrasting functions are the result of macrophage functional plasticity, since they change their phenotype in response to local microenvironment cues [26]. Thus, macrophage activation involves a complex interplay between infiltrated immune cells, resident damage cells and apoptotic cells orchestrated by a number of cytokines/chemokines and growth factors.

Traditionally, in vitro studies have classified Mφ as classically activated Mφ (M1) and alternatively activated Mφ (M2) based on the activation mechanism and cell function [27]. The M1 phenotype is triggered by microbial molecules or inflammatory cytokines, such as lipopolysaccharide (LPS) and interferon gamma (IFN-γ) and releases proinflammatory cytokines and cytotoxic mediators. Accordingly, M1 is involved in the initiation phase of inflammation and is related to tissue damage and proinflammatory functions. In contrast the M2 phenotype produces anti-inflammatory cytokines, growth factor and proangiogenic cytokines involved in the wound healing process (repair phase). Therefore, after the injury, if the interaction between inflammation and repair is well structured, the normal state of the tissue is restored. However, if the initial insult remains perpetuating the inflammation or if the wound healing process is deregulated, it can cause fibrosis [28]. This dichotomous M1/M2 classification of macrophage remains widely used, although represents a simple overview of the macrophage phenotype and function, since the tissue microenvironment has a rather complex combinations of stimuli. For instance, kidney resident Mφ characterized as F4/80^+^ CD64^+^ CD11b/c^int^ were able to limit kidney injury through angiogenesis and wound healing while expressing both pro- and anti-inflammatory/fibrotic genes in a murine model of renal artery stenosis [29]. In another model of chronic kidney damage, known as unilateral ureteral obstruction (UUO), Sogawa et al. revealed that an impaired M1 infiltration suppressed fibrosis and inflammation after UUO in Nrf2 deficiency mice, implying that inflammasome activation can cause chronic inflammation and renal fibrosis through the M1 phenotype [30]. Recently, UUO-induced renal fibrosis was found ameliorated in a myeloid-specific RBP-J deficient macrophage model were both phenotypes (M1 and M2) were compromised by the Notch blockade, suggesting that Notch regulates macrophage phenotype outside the conventional M1/M2 classification [31].

In addition to the Mφ response to cytokine cues, other mechanisms have been determined. Zhang et al. exposed that a triggering receptor expressed on myeloid cells 1 (TREM-1) modulates the Mφ phenotype towards M1 under high-glucose in vitro conditions; in conjunction the expression of TREM-1 in the renal interstitium is significantly correlated with the DN progression in human renal biopsies [32]. A recent study has shown that pioglitazone, a peroxisome proliferator activated receptor γ (PPARγ) agonist, promotes the M2 polarization; although it indicated that the process was not mediated by PPARγ, pioglitazone treatment in M2 cells did increase the expression of vascular endothelial cell growth factor receptor-3 (VEGFR3) via a PPARγ-dependent pathway [33]. Furthermore, VEGFR3 binds to vascular endothelial cell growth factor-C (VEGF-C) and VEGF-C has been identified to ameliorate renal interstitial fibrosis through lymphagiogenesis in UUO [34]. In another in vitro study, it has been established that advanced oxidation proteins products (AOPP) induces the transition of Mφ into dendritic-like-cells and reduces the cell surface thiol pool. Moreover, this effect was lost when the cells were pretreated with the antioxidant N-acetyl cysteine before AOPP exposure. Thus, it indicates that in an oxidizing environment, uremic toxins like plasma AOPP modifies the Mφ response by altering the thiol redox equilibrium [35].

The epithelial-to-mesenchymal transition (EMT) of tubular cells is also a feature of renal fibrosis. During EMT, epithelial markers are lost and mesenchymal genes overexpressed, consequently epithelial cells undergo phenotypic change, some involving myofibroblast transformation with the consequent extracellular matrix accumulation. Although the origin of myofibroblasts in fibrosis is still on debate, it seems that they were derived from residents cells that migrate, proliferate and transform. Some lineage-tracing studies have determined endogenous stromal cells (fibroblasts and pericytes) as the primary source [36,37], and tubular epithelium undergoing EMT as a plausible source in advanced stages where the basement membrane have suffered severe damage [38]. Nonetheless, subsequent studies have determined that Mφ are the main participants as revealed by the macrophage-to-myofibroblast transition occurring in active renal fibrosis [39,40], with the M2 phenotype being the predominant cell type [41]. Nevertheless, when Mφ becomes anti-inflammatory they support the resolution of inflammation. This process involves the expression of growth factors that promote both parenchymal and mesenchymal repair; these is the case of the anti-inflammatory cytokine TGF-β1 that activates mesenchymal repair mechanisms, but in addition to this is a profibrotic cytokine [42]. At the later stage after injury, Mφ contributes to the removal of fibrous tissue. Mediating the resolution phase of healing includes capillary regression and collagen remodeling. In this phase, Mφ can produce factors that terminate the repair response.

## 3. Macrophages and Diabetic Nephropathy

Mφ plays a significant role in the development of DN and is the cause of renal tissue stromal hyperplasia and irreversible pathological changes of glomeruli [43]. In experimental models of diabetes, it has been found that Mφ infiltration occurs at an early stage of the disease and correlates with renal injury. In addition, therapeutic strategies targeted to reduce monocyte infiltration attenuate the development of kidney injury [44].

Mφ depletion using diphtheria toxin (DT) in the CD11b–DT receptor (CD11b-DTR) transgenic mice confirmed the direct role of these cells in DN progression. This study showed that treatment with DT in order to deplete Mφ after induction of diabetes significantly reduced albuminuria, kidney macrophage recruitment and glomerular histological changes [45].

Increased expression of ICAM-1 by renal tubular cells responding to high circulating levels of glucose and advanced glycation end products (AGEs) in diabetic kidney enhances the recruitment of Mφ [46,47]. Once recruited, Mφ responds to local high levels of glucose, AGEs and oxidized low-density lipoprotein (Ox-LDL) to execute inflammatory cytokine secretion. Stimulation of the production of reactive oxygen species (ROS) and proteases continuously provoke Mφ stimulation, leading to tissue damage and renal fibrosis [12].

**Figure 1:**
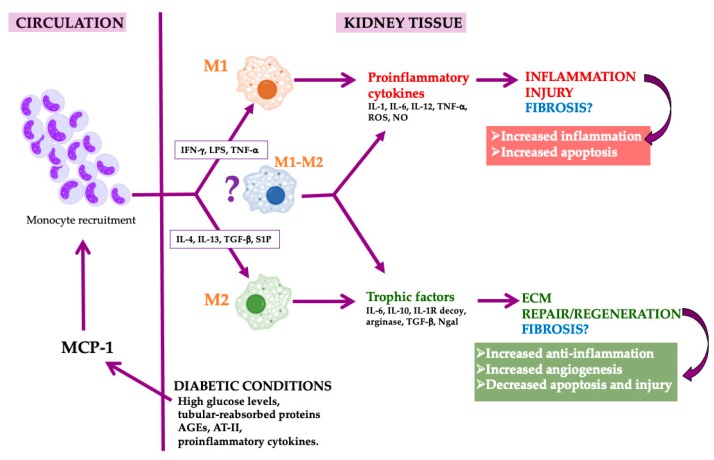
Macrophages in diabetic nephropathy. High glucose levels, tubular-reabsorbed protein, advanced glycation end products (AGEs), angiotensin-II (AT-II) and proinflammatory cytokines induce the production of MCP-1 by kidney tubular cells and podocytes. As a consequence, kidney monocyte recruitment is provoked. In the tissue, monocytes are converted in macrophages that acquire the M1, M2 or mixed phenotype depending on the inflammatory milieu and the molecules released by these different macrophage populations. M1 provokes injury and M2 are proresolution and regenerative macrophages that could induce fibrosis, while mixed macrophages could acquire different roles depending on the milieu. Macrophage image by Mikael Häggström, used with permission.

A key mediator in the infiltration of Mφ is the monocyte chemoattractant protein-1 (MCP-1) [48] (Figure 1). In fact, MCP-1 deficient diabetic mice do not develop albuminuria and is protected from an increase in plasma creatinine [49]. Additionally, urinary MCP-1 is upregulated in inflammatory renal disease and diabetic nephropathy [50]. Upregulated renal MCP-1 levels released from kidney tubular cells, smooth muscle cells, mesangial cells and podocytes are induced by elevations in glucose levels, tubular-reabsorbed protein, AGEs, angiotensin-II (AT-II) and in response to proinflammatory cytokines such as interleukin-1, TNF-α and interferon-γ [45]. In addition to Mφ accumulation, MCP-1 is thought to be indirectly involved in the recruitment of neutrophils through an intermediate mediator leukotriene B4 [51].

The P2X7 receptor is expressed on Mφ and mediates proinflammatory signaling pathways. In human mesangial cells exposed to a high glucose environment, P2X7 receptors have increased the release of MCP-1 [52]. Furthermore, mice with a knockdown of the P2X7 receptor had reduced glomerular macrophage recruitment and collagen IV deposition. The use of NOX-E36 (emapticap pegol), which binds and inhibits MCP-1, resulted in a renal function improvement in patients with type 2 diabetes [53].

MCP-1 binds to C–C chemokine receptor 2 (CCR2) and treatment with the CCR2 antagonist CCX140-B resulted in decreased albuminuria, lessen glomerular hypertrophy and increased podocyte density in transgenic human CCR2 knockin mice rendered diabetic [54]. In addition, CCX140-B treatment in patients with type 2 diabetes and nephropathy stemmed a reduced albuminuria, highlighting their renoprotective role when given in addition to standard care [55]. Phase 3 clinical trials are now planned for the CCR2 antagonist CCX140-B.

Thus, it seems that reduction of Mφ recruitment by the use of agents able to reduce its accumulation in diabetic kidneys, improves renal function and fibrosis. In this sense, therapeutic approaches with beneficial renal outcomes in DN can also focus in antibodies against proteins involved in macrophage infiltration (Table 1).

In addition, Mφ could be activated by inflammatory mediators released by activated lymphocytes. Activated T lymphocytes could secrete IFN-γ and/or TNF-α, both activating macrophages and inducing renal fibrosis [62]. It is known that cell adhesion molecules and inflammatory cytokines provoke the migration of T lymphocytes to the kidney and abnormal activation and migration of T lymphocytes contributed to the development of renal fibrosis in DN [62]. Pharmacological maneuvers able to provoke a reduction in the infiltration of T lymphocytes by inhibiting the production of MCP-1 and the proliferation of T lymphocytes, but promoting their apoptosis in diabetic kidneys of animal models or DN, induces anti-inflammation and antioxidant effects and finally reduces proteinuria and renal fibrosis [63].

In contrast with the above findings, pointing to a harmful role of lymphocytes in DN, there is also evidence that accumulation of IL-17A producing T helper cells (Th17 cells) in diabetic kidneys may help to limit the progression of diabetic nephropathy [64,65]. In this sense, mice with an IL-17A gene defect develop more serious renal injury of DN, while wild-type (WT) diabetic mice receiving a low dose of IL-17A are protected against DN [64]. Notably, the IL-17A treatment was related to the reduction of the Mφ infiltration, proinflammatory cytokines (MCP-1, IL-10, IL-6 and TNF-α) and STAT3 activation, thus revealing an anti-inflammatory effect in diabetic mice. Moreover, in diabetic patients, the urine level of IL-17A was increased in the presence of microalbuminuria but decreased in the presence of macroalbuminuria [65]. Therefore, IL-17A can protect DN by alleviating the inflammatory response.

Another study assessed the role of T-regulatory cells in the development of diabetic nephropathy in *db/db* mice. This study showed that depleting T-regulatory cells with anti-CD25 mAb could exacerbate diabetic renal injury and adoptively transferring CD4+FoxP3+cells into mice could reduce diabetic renal injury [66].

## 4. Macrophage Phenotype and Diabetic Nephropathy

As mentioned, macrophage plasticity allows them to acquire different phenotypes [67]. M1 and M2 play opposing roles since M1 is important for antigen presentation and immune inflammatory effects, whereas M2 mainly release cytokines that inhibit inflammation and exert anti-inflammatory effects and proresolution mechanisms [68].

Mφ located at the site of diabetic kidney injury are predominantly M1. The relevance of M1 to the development of DN has been shown using mice with Mφ cyclooxygenase-2 (Cox-2) deletion. These mice demonstrate increased M1 polarization, which is associated with increased renal injury [69]. Although other authors found that Mφ in the kidneys of streptozotocin-induced DN rat are characterized by an elevated expression of galectin-3 and TGF-β, suggesting an M2 dominance [59]. Adoptive transfer of proresolution M2 to streptozotocin-induced type 1 diabetic mice led to decreased macrophage infiltration to the kidney along with decreased renal damage including tubular atrophy, glomerular hypertrophy and interstitial expansion [70]. Agents that promote M2 polarization like Pentraxin-3 are able to attenuate renal damage in DN by promoting M2 macrophage differentiation [58]. Zhang et al. suggested that the inhibition of M1 macrophage activation and the promotion of M2 macrophage transformation prevented podocyte injury [71]. Similarly, activation of M2 by Sirt6 protected podocytes against injury in a mimicked diabetic kidney microenvironment [72].

In addition, the activated microglia/macrophage WAP (AMWAP) domain protein, known to have anti-inflammatory activity by turning macrophages to the M2 phenotype, protects the kidney against diabetic nephropathy, reduced albuminuria and glomerulosclerosis possibly through regulation of the macrophage phenotype [64].

However, enalapril treatment causing a repolarization of the macrophage towards a M1-like phenotype appears to inhibit the progression of kidney damage in DN experimental models [59].

Hence, beneficial kidney macrophage in the diabetic kidney may be similar to a phenotype described as a resolution macrophage, but the substances that define this proresolution phenotype are not established. These resolution Mφ do not always express markers that characterize them as either classically nor alternatively activated but are a hybrid of both phenotypes. Further studies will be necessary to characterize the functions of Mφ in the diabetic kidney. For example, although increased COX-2 expression in Mφ is often cited as a characteristic of a proinflammatory, M1 phenotype, other studies indicate that its expression may actually mitigate against detrimental effects in DN [73]. It has been found that administration of a heme oxygenase suppressed M1 and restored M2 in association with decreases in proinflammatory cytokine/chemokine, reduction of extracellular matrix/profibrotic protein and improvement of kidney function and histology [73]. These restoring macrophages are characterized by increased COX-2 expression, and maintenance of this phenotype is dependent upon macrophage-derived cAMP production. These data suggest that therapeutic strategies, which reduce the M1 phenotype and promote the proresolution phenotype in the kidney could have significant potential in the treatment and management DN. The time course of the different phenotypes is not clear.

In general, in the process of kidney fibrosis, first, resident immune cells, including Mφ produce chemoattractant substances that enhance inflammatory responses by recruiting more M1, after that, the change in tissue environment shapes the macrophage phenotype towards M2, with functional properties that meet the tissues need to address the danger. At the later stage after injury, M2 contribute to the removal of fibrous tissue by phagocytosis, mediating the resolution phase of healing that includes collagen remodeling. However, these healing M2 could become profibrotic, but little is known about how this particular population of Mφ terminates the repair response and becomes profibrotic. In experimental models of kidney fibrosis, comparing different M2 therapies, it has been found that not all M2 therapies were effective, only therapies using genetically modified Mφ, able to maintain a stable M2 phenotype were effective. The rest of M2 therapies become at some point profibrotic if they do not have a stable phenotype and were not antifibrotic [74]. These genetically modified Mφ overexpressed neutrophil gelatinase associated lipocalin (NGAL) being genetically stable and capable of preserving their anti-inflammatory and antifibrotic phenotype even when placed in a proinflammatory and profibrotic environment.

## 5. Macrophages Phenotype under the Control of Bioactive Molecules Generated in the Kidney

Apart from the classical process of activation and recruitment of M1 that initiate the inflammatory process, depending on the degree of inflammation and/or the environment, Mφ can become proresolution cells. These proresolution Mφ could become profibrotic and moreover, there are Mφ with mixed phenotypes that will also be part of these populations.

Depending on what Mφ synthesize or express and on the molecules of the environment capable of modifying its secretome, they can change the phenotype and express mixed phenotypes or profibrotic phenotypes.

Among the bioactive molecules capable of changing the phenotype, sphingosine 1-phosphate (S1P) has been described. Sphingolipids are pleiotropic regulators of cellular physiology that modulate diverse pathways of cell death, inflammation and immunity. Phosphorylation of sphingosine by sphingosine kinases (SK1) leads to the formation of S1P. Long term hyperglycemia and oxidative stress could activate SK1 and increase the production of S1P, eliciting expression of proinflammatory adhesion molecules in endothelial cells, vascular smooth muscle cell and glomerular mesangial cell proliferation. Living in the environment of hyperglycemia and oxidative stress, as occurs in diabetes, for a long time, activate SK1 and increase S1P synthesis [75]. Yagobian et al. demonstrates that inhibitors of the formation of S1P reduce tubulointerstitial renal inflammation and fibrosis in diabetic nephropathy [76]. By the contrary, other studies have shown that FTY720, a S1P agonist selectively inhibits lymphocyte migration and alleviates ischemia–reperfusion injury after renal fibrosis by reducing the release of ECM and reducing the progression of glomerulosclerosis [77]. Thus, while the increased expression of S1P under diabetic conditions seems clear, results on its involvement in diabetes progression does not seem so clear and its potential involvement in macrophage phenotype under diabetic conditions remains unexplored.

In inflammation associated to kidney ischemia reperfusion injury, we evidenced that apoptotic cell-derived S1P or exogenously administered S1P analogue FTY720 activates Mφ to support the proliferation and healing of renal epithelium trough the production of NGAL. Both suppression of inflammation and renal regeneration might require S1P receptor 3 (S1P3) signaling and downstream release of NGAL from Mφ [78]. Despite being controversial, S1P may increase the production of proresolution Mφ. It is not known in fibrosis associated to diabetic nephropathy whether this mechanism operates or is overcome or altered by others.

NGAL is also involved in the development of the kidney, inducing the differentiation of kidney progenitors in the metanephric mesenchyme into renal epithelia [79]. NGAL can be produced in multiple organs, including kidney, liver, heart, gut and various populations of immune cells, such as Mφ or dendritic cells [80,81] and may also be one of the key potential modulators of macrophage phenotype.

Systemic delivery of NGAL has been shown to have protective effects in ischemic AKI by inhibition of tubular cell death and induction of antioxidant genes [82]. NGAL plays a crucial role in epithelial proliferation by attenuating activation of PPARγ and in the induction of epithelial marker expression via activating megalin and the downstream activation of PI3K/Akt signaling pathway [83].

In addition, Mφ overexpressing NGAL could induce intrinsic resistance to ischemia, causing protection from kidney ischemia–reperfusion injury [84]. Moreover, IL-10–overexpressing Mφ could protect kidney from ischemic AKI and improve tissue repair through the induction of NGAL [85]. Similar to this study, it has been found that NGAL KO mice had worse renal damage after IR-induced AKI [86]. Furthermore, NGAL therapy is effective in DN [87]. In this study, macrophage NGAL cell therapy increased the anti-inflammatory molecule IL-10 and decreased the proinflammatory molecule TNF-α, indicating that NGAL overexpressing macrophage therapy could reduce inflammation in DN. This anti-inflammatory effect may have both a direct therapeutic effect on DN and at the same time could contribute to M2 phenotype stabilization.

The NLRP3 inflammasome is the best studied among the inflammasomes, multiprotein cytosolic complexes that form part of the NLR family of PRR [88,89]. NLRP3 is involved in chronic kidney disease pathogenesis [90,91]. In tissue from human renal biopsies, increased expression of NLRP3 mRNA was detected in diabetic kidney diseases. In addition, NLRP3 was associated with renal function and inhibition of NLRP3 inflammasome activation prevents renal inflammation and fibrosis at least in part via suppression of oxidative stress in diabetic nephropathy [92].

Activation of NLRP3 inflammasome plays a critical role in macrophage M1 polarization [93,94]. NF-κB activation is a hallmark of M1 and it has been shown that it promotes renal injury in crescentic glomerulonephritis [95]. Additionally, if NF-κB activation is inhibited in Mφ, they acquire an anti-inflammatory phenotype and suppress renal injury when transferred it in a rat model of nephrotoxic nephritis [96]. Conversely, NLRP3 is also involved in M2 polarization since NLRP3 transactivate IL-4 promoter to increase IL-4 expression (characteristic of M2 phenotype) in peripheral blood monocyte-derived Mφ [97]. In addition, it has been found in UUO mice that NLRP3 inflammasome activates NF-κB and IL-4/STAT6 signaling pathways and promotes M1 and M2 macrophage infiltration and renal injury [98]. Hence, indicating that targeting NLRP3 inflammasome in diabetes could act on Mφ in diabetic nephropathy.

## 6. Conclusions

Although the role of the different Mφ phenotypes in fibrosis and the effectivity of Mφ therapies with a stable M2 phenotype (genetically modified) were studied, it remains unclear how a M2 phenotype could become profibrotic and what is the influence of the environment.

Factors that modulate S1P, NGAL, or NLPR3 could be effective in the context of diabetic nephropathy, because of its ability to modify Mφ phenotype or the ratio between proresolution and inflammatory Mφ.

## Figures and Tables

**Table 1:** Diabetic nephropathy therapies with macrophage implications.

Drug/mAbs	Mechanism of Action	Macrophage Implication	Outcome	Ref.
**Alantolactone**	Inhibition of TNF-α and IL-6	Reduces Mφ infiltration	In diabetic mice: reduced creatinine and urea nitrogen serum levels.	[56]
**Tectorigenin**	Improve vascular endothelium dysfunction through AdipoR1/2 pathway	Reduces Mφ infiltration and M1 polarization	In diabetic mice: reduced endothelia damage through lipotoxicity, improved insulin sensitivity, attenuated Mφ-induced inflammation	[57]
**Emapticap Pegol**	Inhibition of MCP-1	Reduces Mφ infiltration	In diabetic patients: reduced HbA1c and urinary albumin/creatinine ratio.	[53]
**Pentraxin-3**	Increase numbers of Mφ expressing Arg1 - CD206	Promotes M2 polarization	In diabetic mice: increased expression of nephrin, acetylated nephrin, and WT-1.	[58]
**Enalapril**	Increase T cells number and promotes Mφ differentiation towards M1-like	Promotes M1-like polarization	In diabetic patients: reduced albuminuria without modulating the HbA1c %.	[59]
**Monoclonal Antibodies Against CD148**	Prevents reduction of podocyte and nephrin expression and decreased glomerular fibronectin expression	Reduces Mφ infiltration	In diabetic mice: decreased albuminuria and mesangial expansion without altering hyperglycemia and blood pressure	[60]
**Monoclonal Antibody against IL-17**	Blocks the NF-κB cascade, TGF-β and fibronectin.	Reduces Mφ infiltration	In diabetic mice: reduced albuminuria, glomerular damage, Mφ accumulation and renal fibrosis	[61]

AdipoR1/2: adiponectin receptor 1/2. WT1: Wilms’ tumor-1 protein. Arg1: arginase 1. CD206: mannose receptor. HbA1c: hemoglobin A1c.
